# Fully automated artificial intelligence–based echocardiographic analysis substantially reduces workflow time while preserving measurement accuracy: a pilot study

**DOI:** 10.1186/s44348-026-00073-w

**Published:** 2026-05-20

**Authors:** Jonghee Sun, Yeonyee E. Yoon, Jiyeon Lee, Ganghan Lee, Minjung Bak, Jiesuck Park, Hong-Mi Choi, In-Chang Hwang, Goo-Yeong Cho

**Affiliations:** 1 Cardiovascular Center and Division of Cardiology, Department of Internal Medicine, Seoul National University Bundang Hospital, Seoul National University College of Medicine, Seongnam, Republic of Korea; 2 Ontact Health Co Ltd, Seoul, Republic of Korea

**Keywords:** Echocardiography, Artificial intelligence, Automation, Ventricular function

## Abstract

**Background:**

Transthoracic echocardiography (TTE) requires time-intensive integration of quantitative measurements and qualitative visual assessment. Fully automated artificial intelligence (AI)-based analysis may reduce total analysis time while preserving accuracy, but systematic real-world validation remains limited.

**Methods:**

This prospective, single-center pilot study enrolled 40 TTE examinations. Identical deidentified DICOM datasets were independently provided to a trained cardiac sonographer and a fully automated AI system comprising quantitative and qualitative visual interpretation modules. All outputs were compared with a cardiologist-adjudicated reference standard. Primary endpoints were total analysis time and noninferiority of AI-derived left ventricular ejection fraction (LVEF) versus the reference standard, with a prespecified margin of 3 percentage points (one-sided α = 0.025).

**Results:**

Median analysis time was 94 s (interquartile range [IQR], 82–106 s) for the AI workflow versus 490 s (IQR, 438–626 s) for the human workflow (P < 0.001). AI-derived LVEF met the noninferiority criterion (mean difference, 0.00 percentage points; upper one-sided 95% confidence bound, 1.41 percentage points; P < 0.001), with an intraclass correlation coefficient (ICC) of 0.902 (95% confidence interval, 0.822–0.947). ICCs for secondary quantitative indices ranged from 0.625 to 0.989. For aortic regurgitation severity grading, AI’s overall accuracy was 75.0% (quadratic weighted κ = 0.762), compared with 82.5% for human interpretation (κ = 0.812, McNemar P = 0.579).

**Conclusions:**

Fully automated AI-assisted TTE analysis substantially reduced total analysis time while maintaining noninferior LVEF accuracy and acceptable performance across secondary quantitative and qualitative indices. These findings support the use of AI as a practical workflow accelerator in routine echocardiography.

**Supplementary Information:**

The online version contains supplementary material available at 10.1186/s44348-026-00073-w.

## Background

Transthoracic echocardiography (TTE) is the most widely used cardiovascular imaging modality in routine clinical practice and provides essential information for the assessment of cardiac structure, function, and hemodynamics [1]. A comprehensive echocardiographic evaluation requires the integration of multiple quantitative measurements, such as chamber dimensions, volumes, and flow velocities, with qualitative visual interpretation of myocardial motion, valvular pathology, and pericardial abnormalities [2]. Despite its central role in cardiovascular care, echocardiographic analysis is inherently operator dependent, often requiring substantial time and expertise to ensure accuracy and consistency across a broad range of parameters. In contemporary clinical environments, increasing imaging volumes and limited human resources further amplify the burden of conventional manual analysis.

Recent advances in artificial intelligence (AI) have enabled automatic quantitative measurements [3, 4] and, in some systems, portions of visual interpretation [5–8]. These technologies have the potential to shorten analysis time while maintaining or improving the accuracy and reproducibility of key TTE indices, thereby serving as practical adjuncts to manual workflows [9–12]. Despite this potential, systematic real-world validation of AI-based automated echocardiographic analysis remains limited. Therefore, we conducted an exploratory pilot study to assess the feasibility of implementing a fully automated AI-based analysis in routine clinical practice and to compare its performance with conventional manual quantitative analysis and visual qualitative assessment. Specifically, we evaluated whether the AI-based approach reduces total analysis time while preserving clinically acceptable accuracy for key echocardiographic outputs.

## Methods

### Ethics statement

This study was approved by the Institutional Review Board of Seoul National University Bundang Hospital (No. B-2601–1023-108). The requirement for written informed consent was waived owing to the retrospective nature of data collection and the use of deidentified DICOM datasets.

### Study design

This study used retrospectively collected TTE examinations and performed a prospective, paired comparison between human and AI analyses under a predefined protocol. For each examination, a trained cardiac sonographer performed conventional manual quantitative measurements and visual qualitative assessment, while the AI system generated outputs through a fully automated workflow without human intervention. Total analysis time was recorded for each approach, and outputs from both methods were compared with cardiologist-adjudicated reference standard (ground truth, GT) to assess the accuracy of key echocardiographic metrics.

### Study population

We screened adult TTE examinations performed at Seoul National University Bundang Hospital between November and December 2025. To enable a paired comparison between fully automated AI-based analysis and human analysis, we selected studies in which a predefined core imaging checklist could be completed. Specifically, eligible examinations were required to include standard B-mode views (parasternal long axis; parasternal short axis views at the levels of great vessels, mitral valve, and papillary muscle; a right ventricular–focused view; and apical four-, two-, and three-chamber views), M-mode recording of the aortic root-left atrium and left ventricle (LV), and Doppler acquisition covering continuous-wave Doppler across the aortic valve, pulsed-wave Doppler of the LV outflow tract, continuous-wave Doppler of tricuspid regurgitation, and septal tissue Doppler imaging. To minimize the risk of data leakage, we excluded TTE studies from patients known to have been included in the Open AI Dataset Project (AI-Hub; https://www.aihub.or.kr/), a national echocardiographic database supported by the Korean Ministry of Science and ICT [9–14]. This exclusion was applied because the AI-based TTE analysis solution evaluated in this study (SONIX Health ver. 2.0, Ontact Health) was developed using data from the prior AI-Hub project, and any overlap could inflate performance estimates. In addition, TTE examinations were excluded when image quality was unmeasurable, such that expert adjudication of key measurements or qualitative assessment for the reference standard could not be performed; if a repeat examination from the same patient was identified during the screening window; or if essential metadata (age, sex, height, or weight) were missing.

The sample size was determined to address two primary aims: superiority in efficiency (shorter total analysis time) and noninferiority in accuracy. LV ejection fraction (LVEF) was selected as the representative quantitative endpoint for the noninferiority analysis. For efficiency, prior literature on manual echocardiographic workflows informed an estimated mean manual analysis time of 9.0 ± 2.0 min for the scope of this study (excluding narrative text entry and formatting) [15]. Assuming a 50% reduction with fully automated AI analysis, a paired t-test (two-sided α = 0.05, power = 80%) yielded a minimum requirement of two-paired examinations. However, the overall sample size was driven by the accuracy endpoint.

For accuracy, a noninferiority framework was prespecified using Simpson biplane LVEF. The primary comparison evaluated the difference between the fully automated AI-derived LVEF and the reference standard, defined as Δ = AI – GT. The noninferiority margin was set at δ = 3 percentage points. Although interobserver variability for Simpson biplane LVEF has been reported to be approximately 7 to 9 percentage points, structured training and educational interventions may reduce residual disagreement to approximately 3 percentage points in standardized settings [16–18]. On this basis, we adopted δ = 3 percentage points as a stringent but clinically meaningful threshold. Using this margin (one-sided α = 0.025, power = 80%) and an assumed standard deviation (SD) of Δ (SD_Δ = 5 percentage points) informed by prior studies [19], the minimum required sample size was estimated at approximately 22 examinations. To account for variability in image quality, potential AI analysis failures, and clinical heterogeneity, we targeted 40 TTE examinations for this exploratory pilot study.

Given the pilot nature of the study and the limited number of cases, we applied purposive sampling to ensure clinical diversity. We intentionally included examinations spanning a wide range of LVEF values, incorporated cases with atrial fibrillation as well as fair-to-poor image quality, and enriched the cohort with aortic regurgitation (AR) across a spectrum of severities to support evaluation of the accuracy of qualitative visual assessment.

### Echocardiography acquisition, reference standard, and data preparation

All TTE examinations were acquired in routine clinical practice by trained cardiac sonographers or cardiologists using standardized institutional protocols consistent with contemporary echocardiographic guidelines [1]. As part of standard clinical reporting, primary quantitative measurements were obtained by the acquiring personnel, and examinations were interpreted clinically by board-certified cardiologists specializing in echocardiography.

For this pilot study, we performed an additional reference-standard adjudication. An experienced cardiologist (YEY, with over 15 years of experience) subsequently reviewed the complete image dataset for each examination, together with the original clinical report and recorded measurements. Key measurements and qualitative grades were verified and, when necessary, remeasured and re-adjudicated in accordance with guideline-based criteria to establish the final reference standard. For AR, severity was adjudicated using a five-category ordinal scale: none, trivial, mild, moderate, or severe. To facilitate consistent grading, intermediate descriptors commonly used in clinical reporting (e.g., mild-to-moderate or moderate-to-severe) were not permitted for the reference standard assessment. Adjudication was performed in a blinded manner, with no access to any AI outputs. Image quality was also assessed for each examination. Because studies deemed unmeasurable were excluded during screening, image quality among included examinations was categorized using a three-level scale: good, average, or poor.

To enable a paired comparison under identical input conditions, the same DICOM image set was prepared for both the fully automated AI analysis and the human analysis. Because the examinations were retrospectively collected and some DICOM objects may contain measurement annotations, overlays, or derived outputs from prior clinical reporting, these DICOM objects were excluded from the analysis dataset. The remaining DICOM series were deidentified and assigned a study code, and the corresponding anonymized DICOM set was then provided separately to the AI system and the human analyst. Both AI processing and human analysis were performed using only deidentified DICOM data.

### AI-based automated analysis system and workflow

The AI solution evaluated in this study comprised two components: an automated quantitative measurement module (SoniX EchoQuant, Ontact Health) and an automated qualitative (visual) interpretation module (SoniX EchoVerse, Ontact Health). The quantitative measurement component is currently commercially available (SONIX Health ver. 2.0) and is installed in routine operation at Seoul National University Bundang Hospital. In contrast, the qualitative visual analysis component was investigational and was used experimentally in this pilot study while undergoing regulatory preparation. Importantly, the investigational qualitative component was included to enable a fair comparison of analysis time with the human workflow; in routine practice, manual measurements are performed concurrently with visual interpretation, and evaluating the AI system using quantitative automation alone would not reflect the full scope of tasks performed during human analysis.

Both components operated in a fully automated manner without human intervention. After automatic view recognition, the system generated outputs when the required views were available, including 75 quantitative indices and 162 qualitative assessment items. The runtime of each module was recorded separately under the same execution environment used in our institution (CPU: 48 cores, Intel Xeon w7-3455, Intel Corp; GPU: NVIDIA RTX A6000, Nvidia Corp). For performance evaluation, accuracy was assessed only for variables corresponding to the items on the standardized core checklist used for human analysis.

### Human echocardiographic analysis workflow

Human analysis was performed using the institution’s cardiology-dedicated PACS system (INFINITT Cardiology PACS, Infinitt Healthcare). The same deidentified DICOM dataset provided to the AI system was loaded into the PACS for human review and manual measurement. A single trained cardiac sonographer (JL, with 6 years of experience) performed the analysis independently, blinded to the original clinical report and all AI outputs. Because the PACS system provides a full set of echocardiography-specific measurement tools, the analyst performed conventional manual quantitative measurements concurrently with visual quality assessment. All manual measurements and visual assessments were recorded using a standardized core checklist that mirrored the structure of the institution’s routine TTE report. The core checklist template is provided in Material S1. The total analysis time for the human workflow was measured by recording the time when the DICOM study was loaded into the PACS and the time when analysis and checklist documentation were completed; the elapsed time between these timestamps was used as the total human analysis time.

### Statistical analysis

Continuous variables are summarized as median with interquartile range (IQR), and categorical variables as numbers and percentages. The primary efficiency outcome was total analysis time per examination. Human and AI analysis times were compared using paired analyses. Paired differences were tested using the Wilcoxon signed rank test, and results are reported as median with IQR. A two-sided P-value of < 0.05 was considered statistically significant for efficiency comparisons.

For the primary accuracy endpoint, agreement between AI-derived LVEF and the cardiologist-adjudicated reference standard was evaluated using a prespecified noninferiority framework. For each examination, the difference was defined as Δ = (AI) − (reference standard, in percentage points). Noninferiority was tested with a margin of δ = 3 percentage points at a one-sided significance level of α = 0.025 and was concluded if the one-sided 95% upper confidence bound of the mean difference remained below δ (equivalently, if the one-sided P-value for H1: mean Δ < δ was < 0.025). Agreement was further summarized using the intraclass correlation coefficient (ICC) for absolute agreement (ICC (2,1)) with 95% confidence intervals (CIs) and Bland–Altman analysis (mean bias and 95% limits of agreement). Error magnitude was reported using absolute error (|Δ|), summarized as mean ± SD and median (IQR).

As supportive benchmarking, human-derived LVEF were compared with the reference standard using the analogous difference Δ = human − reference standard for continuous variables and the same agreement metrics for categorical variables. These human – reference standard comparisons were intended to provide clinical context and were not used for the prespecified primary noninferiority hypothesis testing.

For secondary quantitative endpoints (e.g., LV volumes and other continuous echocardiographic indices), agreement between AI outputs and the reference standard was summarized using ICC (2,1) for absolute agreement with 95% CIs, together with mean difference (bias) and absolute error (reported as mean ± SD and median [IQR]). Bland–Altman analysis was performed for selected key quantitative variables to characterize systematic differences and dispersion.

For secondary qualitative endpoints (e.g., AR severity grade), agreement between AI outputs and the reference standard was evaluated using overall accuracy (exact agreement) and, where applicable, within-one-grade agreement. Intermethod agreement for ordinal grades was quantified using quadratic weighted κ. For supportive benchmarking, the same metrics were calculated for human interpretations versus the reference standard, and paired differences in exact-grade accuracy between AI and human were assessed using McNemar test. All statistical analyses were performed using R ver. 4.5.1 (R Foundation for Statistical Computing).

## Results

### Baseline characteristics

Baseline characteristics of the study cohort are summarized in Table 1. A total of 40 TTE examinations were included. The median age was 73.5 years (IQR, 62.0–83.0 years), and 12 patients (30.0%) were male. Body surface area was 1.69 m^2^ (IQR, 1.56–1.85 m^2^). Sinus rhythm was present in 27 examinations (67.5%), whereas atrial fibrillation was observed in 13 (32.5%). Image quality was rated as good in 19 examinations (47.5%), average in 17 (42.5%), and poor in 4 (10.0%). LV end-diastolic and end-systolic volumes were 83.0 mL (IQR, 71.5–127.0 mL) and 38.5 mL (IQR, 26.5–62.5 mL), respectively. LVEF was > 55% in 28 examinations (70.0%), 35%–55% in 9 (22.5%), and < 35% in 3 (7.5%). The study cohort was enriched for AR, with AR severity graded as none or trivial in 19 examinations (47.5%), mild in 10 (25.0%), moderate in 8 (20.0%), and severe in 3 (7.5%).

**Table 1:** Baseline characteristics (*n* = 40)

Characteristic	Value
Age (yr)	73.5 (62.0–83.0)
Male sex	12 (30.0)
Body surface area (m^2^)	1.69 (1.56–1.85)
Rhythm	
Sinus rhythm	27 (67.5)
Atrial fibrillation	13 (32.5)
Image quality	
Good	19 (47.5)
Average	17 (42.5)
Poor	4 (10.0)
LV end-diastolic dimension (mm)	49.0 (43.0–55.5)
LV end-systolic dimension (mm)	33.0 (28.0–42.0)
LV end-diastolic volume (mL)	83.0 (71.5–127.0)
LV end-systolic volume (mL)	38.5 (26.5–62.5)
LVEF (%)	59.1 (51.9–63.5)
< 35	3 (7.5)
35–55	9 (22.5)
> 55	28 (70.0)
E velocity (m/sec)	0.71 (0.58–0.92)
A velocity (m/sec)^a^	0.74 (0.59–0.90)
Deceleration time (msec)^a^	195.36 (168.76–244.17)
s' septal (cm/sec)^a^	5.80 (4.75–7.15)
e' septal (cm/sec)	6.10 (4.45–7.30)
a' septal (cm/sec)	8.70 (7.50–10.70)
AR severity	
None or trivial	19 (47.5)
Mild	10 (25.0)
Moderate	8 (20.0)
Severe	3 (7.5)

Values are presented as median (interquartile range) or number (%)

AR, aortic regurgitation; LV, left ventricular; LVEF, left ventricular ejection fraction

^a^Measured only in examinations with sinus rhythm (n = 27)

### Analysis time and efficiency

**Fig. 1:**
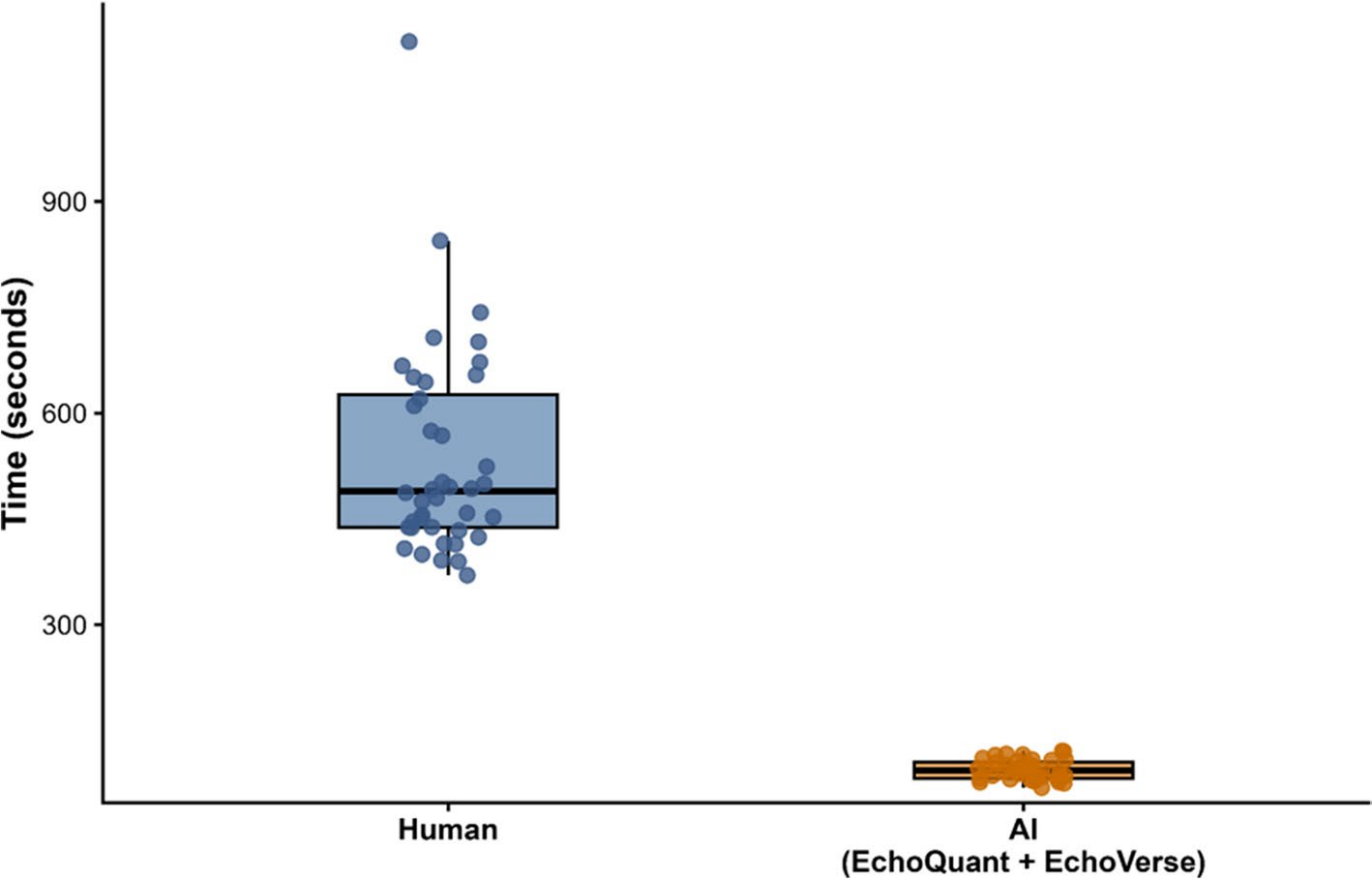
Human versus artificial intelligence (AI) analysis time. Box-and-jitter plots show total analysis time per examination for the human workflow and the fully automated AI workflow (EchoQuant + EchoVerse) across 40 paired transthoracic echocardiography examinations. Each dot represents one examination. Boxes denote the median and interquartile range, and whiskers indicate the data range. Total analysis time differed significantly between workflows (Wilcoxon signed rank test, P < 0.001)

All 40 examinations were successfully analyzed by both the human reader and the fully automated AI system, with no analysis failures for either quantitative measurements or qualitative assessment. Human analysis time had a median of 490 s (IQR, 438–626 s; range, 370–1,127 s), whereas the fully automated AI workflow required 94 s (IQR, 82–106 s; range, 69–121 s). When the AI pipeline was examined by modules, EchoQuant required 74 s (IQR, 68–83 s; range, 54–98 s), and EchoVerse required 18 s (IQR, 15–22 s; range, 12–37 s). In paired comparisons, total analysis time differed significantly between the human and AI workflows (Wilcoxon signed rank test, P < 0.001) (Fig. 1).

### Accuracy of key echocardiographic measurements and assessments

#### Primary accuracy endpoint: LVEF noninferiority

**Fig. 2:**
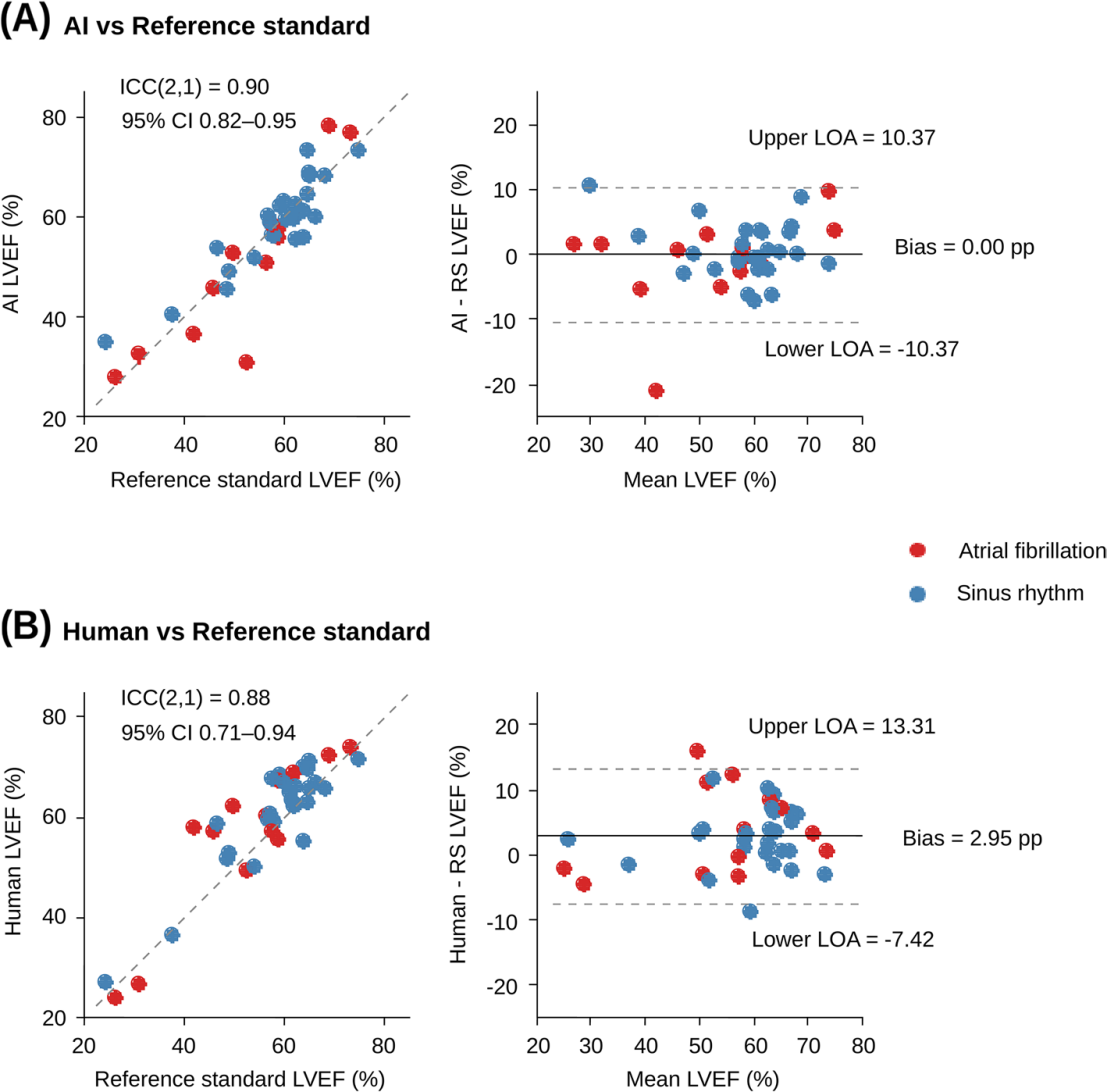
Agreement of left ventricular ejection fraction (LVEF) with the reference standard. (A) Scatter plot of AI-derived versus reference-standard LVEF. (B) Bland–Altman plot for AI-derived versus reference-standard LVEF. (C) Scatter plot of human-derived versus reference-standard LVEF. (D) Bland–Altman plot for human-derived versus reference-standard LVEF. In panels A and C, the identity line is shown. In panels B and D, the solid line indicates mean bias and dashed lines indicate 95% limits of agreement (LOAs). Red dots indicate atrial fibrillation, and blue dots indicate sinus rhythm. Intraclass correlation coefficient (ICC) for absolute agreement (ICC [1, 2]) and Bland–Altman summary statistics are annotated within each plot

For the primary accuracy endpoint, AI-derived LVEF demonstrated noninferiority to the reference standard using the prespecified margin of δ = 3 percentage points (n = 40). The mean difference was 0.00 ± 5.29 percentage points, and the one-sided 95% upper confidence bound of the mean difference was 1.41 percentage points, remaining below the noninferiority margin (P < 0.001) and confirming noninferiority. Agreement between AI-derived LVEF and the reference standard was high, with an ICC (2,1) for absolute agreement of 0.90 (95% CI, 0.82–0.95) (Fig. 2A and B). Bland–Altman analysis showed no systematic bias (bias, 0.00 percentage points) with 95% limits of agreement from –10.37 to 10.37 percentage points. The mean absolute error was 3.53 ± 3.90 percentage points, and the median absolute error was 2.53 percentage points (IQR, 1.02–4.57 percentage points).

As supportive benchmarking, we also compared human-derived LVEF with the reference standard. Human-derived LVEF tended to be higher than the reference standard (mean Δ, 2.95 percentage points; 95% CI, 1.26–4.64; SD, 5.29) with an ICC (2,1) of 0.88 (95% CI, 0.71–0.94) (Fig. 2C and D). The mean absolute error was 4.67 ± 3.81 percentage points, and the median absolute error was 3.43 percentage points (IQR, 2.08–6.73 percentage points).

#### Secondary quantitative endpoints

Agreement between AI-derived quantitative indices and the reference standard was evaluated for variables with available paired data (n = 39 or n = 40). Overall, agreement was high for most measures, with ICCs ranging from 0.625 to 0.989 (Material S2). Doppler- and volume-based parameters showed particularly strong agreement (e.g., E velocity ICC, 0.989; LV end-systolic volume ICC, 0.950; LV end-diastolic volume ICC, 0.940; and left atrial volume ICC, 0.9333). In contrast, comparatively lower agreement was observed for wall-thickness measurements. Across variables, absolute error and mean bias were additionally summarized (Material S2).

#### Secondary qualitative endpoint: AR severity

**Fig. 3:**
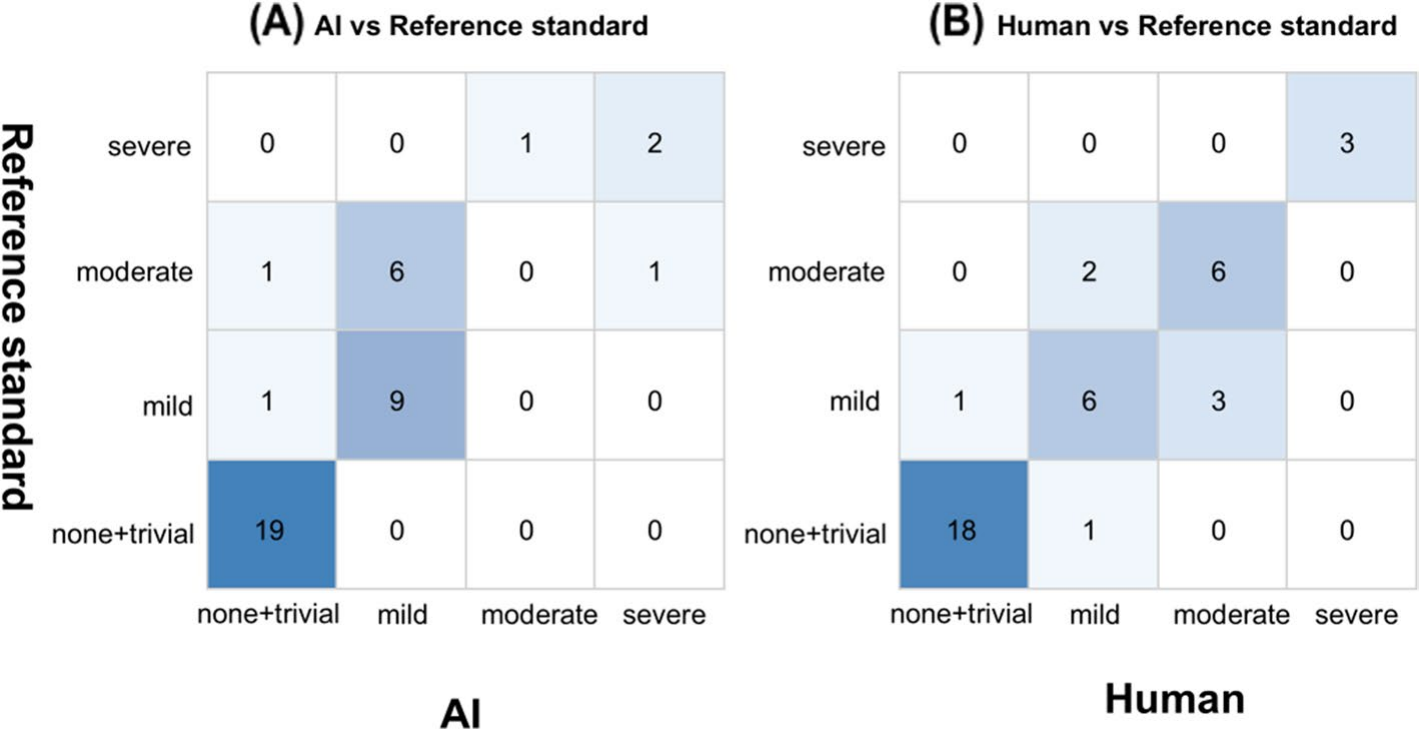
Confusion matrices for aortic regurgitation (AR) severity grading. (A) Confusion matrix comparing artificial intelligence (AI)-predicted AR severity with the reference standard across 40 examinations (overall accuracy, 75.0%; quadratic weighted κ = 0.762). (B) Confusion matrix comparing human-assigned AR severity with the reference standard across 40 examinations (overall accuracy, 82.5%; quadratic weighted κ = 0.812). Rows indicate reference-standard grades and columns indicate predicted grades. Cell shading represents the proportion of examinations in each cell

Agreement between AI-predicted AR severity and the reference standard was moderate to substantial, with an overall accuracy of 75.0% and a quadratic weighted κ of 0.762 (n = 40) (Fig. 3A). Class-wise performance showed high specificity across categories, while sensitivity varied by severity grade. In particular, sensitivity was 1.00 for none/trivial, 0.90 for mild, 0.00 for moderate, and 0.67 for severe AR, yielding a balanced accuracy of 0.642. Detailed class-wise performance metrics (sensitivity, specificity, positive predictive value, and negative predictive value) are provided in Material S3.

For supportive benchmarking, human interpretation demonstrated an overall accuracy of 82.5% with a quadratic weighted κ of 0.812 (Fig. 3B). Sensitivity was 0.947 for none/trivial, 0.60 for mild, 0.75 for moderate, and 1.00 for severe AR, with a balanced accuracy of 0.824. In paired comparisons of exact-grade accuracy against the reference standard, AI and human interpretations agreed in most cases; both were correct in 25 cases (62.5%), both were incorrect in 2 (5.0%), AI alone was correct in 5 (12.5%), and human alone was correct in 8 (20.0%). The difference in exact-grade accuracy between AI and human was not statistically significant (McNemar test, P = 0.579). Additional qualitative grading results for mitral regurgitation, tricuspid regurgitation, and pericardial effusion are provided in Material S4.

## Discussion

In this pilot paired-comparison study using identical deidentified DICOM inputs, a fully automated AI-based echocardiographic workflow substantially reduced total analysis time compared with conventional human analysis, while preserving accuracy for the prespecified key quantitative endpoint, LVEF. All included examinations were successfully processed by both approaches, and the AI workflow achieved a marked reduction in median analysis time despite encompassing both quantitative measurement and qualitative visual assessment. These findings suggest that AI may serve not only as an automated measurement tool, but also as a practical workflow solution for routine echocardiographic analysis.

The most immediate implication of our findings is the potential of AI to alleviate the growing operational burden in contemporary echocardiographic laboratories. Routine TTE interpretation requires a combination of repetitive quantitative measurements and integrative visual assessment [1, 2, 20]. The former is often manual and operator dependent, whereas the latter relies on integrative interpretation of multiple imaging findings in a clinical context. Prior studies have shown that AI can improve efficiency in the echocardiographic post-acquisition workflow by reducing the time required for automated measurement and report generation, but these approaches have largely focused on quantitative tasks rather than integrated visual interpretation [15, 17]. Because manual measurements and visual interpretation are ordinarily performed together in clinical practice, the inclusion of both EchoQuant and EchoVerse allowed a more realistic assessment of workflow efficiency. In our study, the human workflow required a median of approximately 8 min per examination, whereas the fully automated AI pipeline completed analysis in approximately 1.5 min.

A key strength of the present study is that the efficiency gain was accompanied by preserved performance for the prespecified primary accuracy endpoint. AI-derived LVEF met the noninferiority criterion relative to the cardiologist-adjudicated reference standard, despite the use of a stringent no-inferiority margin of only 3% [16–18], and showed essentially no systematic bias and high absolute-agreement ICC. Given that LVEF remains one of the most widely used and clinically consequential summary indices in TTE, this finding supports the practical credibility of automated analysis. Notably, the human reader showed a tendency toward overestimation of LVEF relative to the reference standard, whereas the AI system did not show directional bias. While no inference can be made regarding broader interobserver patterns from a single reader, this result highlights the potential for reader-dependent directional bias in human analysis. Although this comparison was intended only as supportive benchmarking rather than formal superiority testing, it suggests that the automated workflow can provide clinically stable estimation of global LV systolic function under standardized input conditions.

Beyond LVEF, the AI system showed generally high agreement with the reference standard for several secondary quantitative indices, particularly Doppler- and volume-based parameters such as E velocity, LV end-systolic and end-diastolic volumes, and left atrial size or volume. These findings support the feasibility of broader quantitative automation in routine TTE analysis. By contrast, ICC values were lower for wall-thickness measurements, including interventricular septal and posterior wall indices. Nevertheless, the absolute differences for these parameters remained small, with median absolute errors and mean differences generally close to 1 mm. This suggests that the lower ICCs may partly reflect the relatively narrow measurement range of wall-thickness variables rather than large absolute disagreement. In addition, such a pattern is not unexpected, as wall-thickness measurements are often more sensitive than chamber volumes or Doppler velocities to subtle differences in border definition, phase selection, image plane alignment, and local image quality [2]. The observed heterogeneity across quantitative variables, therefore, provides a realistic picture of current performance.

For qualitative assessment, the AI system demonstrated promising but nonuniform performance in AR severity grading. Overall agreement was substantial, and exact-grade accuracy was reasonably high, but class-wise sensitivity varied considerably across severity categories, with particularly limited sensitivity for moderate AR. This likely reflects the intrinsic challenge of grading valvular regurgitation using discrete ordinal categories, especially in intermediate cases where findings may overlap and even expert interpretation relies on integrating multiple semiquantitative cues. In addition, the study design required assignment to a single severity grade and did not allow range-based interpretations, such as mild-to-moderate or moderate-to-severe, which are commonly used in clinical practice and may have further reduced sensitivity in borderline cases. Human interpretation also showed imperfect class-wise sensitivity, although overall performance was somewhat higher. However, these findings should be interpreted in the context of the study design: the EchoVerse module used here was an early developmental version, incorporated primarily to enable a fair time comparison within the fully automated workflow, rather than for formal, standalone validation of qualitative performance. As such, the present results should be regarded as preliminary, while ongoing refinement of the qualitative module may further change its performance characteristics.

Our study extends prior work on AI-assisted echocardiography by evaluating a fully automated workflow under paired and standardized input conditions, rather than focusing primarily on automated quantitative tasks alone. By incorporating both quantitative and qualitative evaluation using the same deidentified DICOM dataset for AI and human review, the study was designed to provide a more realistic view of how AI adoption may influence routine clinical echocardiographic workflow. In this context, the greatest clinical value of such a workflow may lie not in replacing expert interpretation, but in redistributing effort within the echocardiographic reporting process. Automated extraction of quantitative indices and preliminary visual assessments could reduce repetitive manual workload, shorten analytic turnaround time, and standardize the initial phase of reporting. This may be particularly relevant in high-volume laboratories, where pressure to be efficient can affect consistency and lead to reader fatigue. Under such a model, human expertise can be focused more selectively on integrative review, discrepancy resolution, complex pathology, and final clinical synthesis.

Several limitations should be acknowledged. First, this was a single-center pilot study with a small sample size, and the cohort was assembled using purposive sampling to ensure diversity of LVEF, image quality, rhythm status, and AR severity. Accordingly, the results should be interpreted as hypothesis-generating rather than definitive estimates of real-world performance. Second, human analysis was performed by a single trained sonographer, and the reference standard was established by adjudication from a single experienced cardiologist. Although this design enabled a controlled paired comparison, it does not capture interobserver variability across multiple human readers. Third, while the AI workflow included both quantitative and qualitative modules, the qualitative component was investigational and the main text presents only selected qualitative results, primarily AR severity, with additional findings provided in the supplement. Broader validation across additional qualitative endpoints and disease categories will be needed. Finally, all examinations were obtained at a single institution using local acquisition practices and a predefined execution environment; generalizability to other centers, vendors, and workflow ecosystems remains to be established. At the same time, these limitations are consistent with the exploratory nature of the present pilot study, which was intended to provide an initial framework for large-scale validation. The effect size and variability estimates obtained from this dataset may also serve as a useful basis for planning future studies, including formal sample size calculations and multicenter study design.

## Conclusions

This pilot study suggests that a fully automated AI-assisted echocardiographic workflow can markedly reduce total analysis time while preserving clinically acceptable performance for key measurements, particularly LVEF. The findings support the potential role of AI as a practical workflow accelerator in routine echocardiography. Larger multicenter studies are warranted to confirm generalizability, define the scope of reliable automation across diverse parameters, and determine best to integrate such systems into real clinical reporting pathways.

## Supplementary Information

Additional file 1: Material S1. Core checklist template used for human analysis. Material S2. Agreement of secondary quantitative echocardiographic indices with the reference standard (AI versus reference standard). Material S3. Class-wise diagnostic performance for aortic regurgitation grading. Material S4. Confusion matrices for additional qualitative grading (mitral regurgitation, tricuspid regurgitation, and pericardial effusion).

## Data Availability

The data that support the findings of this study are available from the corresponding author upon reasonable request.

## Abbreviations

AI: Artificial intelligence
AR: Aortic regurgitation
CI: Confidence interval
GT: Ground truth
ICC: Intraclass correlation coefficient
LV: Left ventricle
LVEF: Left ventricular ejection fraction
SD: Standard deviation
TTE: Transthoracic echocardiography
