# Supplementary material for: Fully automated artificial intelligence–based echocardiographic analysis substantially reduces workflow time while preserving measurement accuracy: a pilot study
Source: J Cardiovasc Imaging. 2026 May 20;34:10. doi: 10.1186/s44348-026-00073-w (PMC13188400; doi:10.1186/s44348-026-00073-w)
Supplement: Supplementary file 1 — Additional file 1: Material S1. Core checklist template used for human analysis. Material S2. Agreement of secondary quantitative echocardiographic indices with the reference standard (AI versus reference standard). Material S3. Class-wise diagnostic performance for aortic regurgitation grading. Material S4. Confusion matrices for additional qualitative grading (mitral regurgitation, tricuspid regurgitation, and pericardial effusion). [file 44348_2026_73_MOESM1_ESM.docx]

**Supplementary Methods 1**. **Core checklist template used for human analysis.**

A standardized core checklist template was provided in Microsoft Excel format to record all manual measurements and visual assessments during the human workflow. The template was designed to closely mirror the structure and ordering of the institution’s routine TTE report, thereby minimizing unnecessary documentation time during manual review.


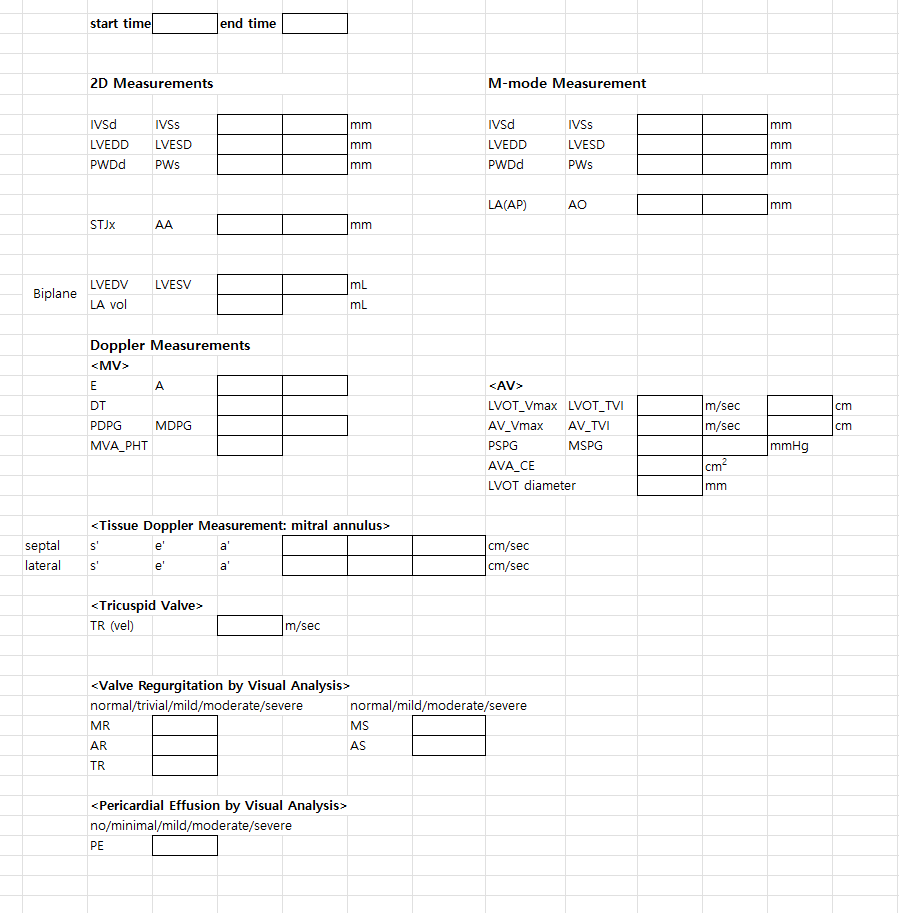


The checklist included dedicated fields for start time (when the de-identified DICOM study was loaded into the cardiology PACS) and end time (when measurements and checklist documentation were completed), from which total human analysis time was calculated. For qualitative visual assessments, dropdown menus were used to enforce consistent grading. Valve regurgitation severity (mitral regurgitation [MR], aortic regurgitation [AR], tricuspid regurgitation [TR]) was recorded using a five-level dropdown (none/trivial/mild/moderate/severe). Valve stenosis severity (mitral stenosis [MS], aortic stenosis [AS]) was recorded using a four-level dropdown (normal/mild/moderate/severe). Pericardial effusion severity was recorded using a five-level dropdown (no/minimal/mild/moderate/severe).

**Supplementary Results 1. Agreement of secondary quantitative echocardiographic indices with the reference standard (AI versus reference standard)**

Secondary quantitative agreement analyses were performed for variables with sufficient paired data, defined as ≥30 examinations with both AI-derived values and reference-standard measurements available. For these variables, agreement was summarized using ICC (2,1) for absolute agreement with 95% confidence intervals, together with the median absolute error (|AI−RS|; median [Q1–Q3]) and the mean difference (Δ = AI−RS).

**
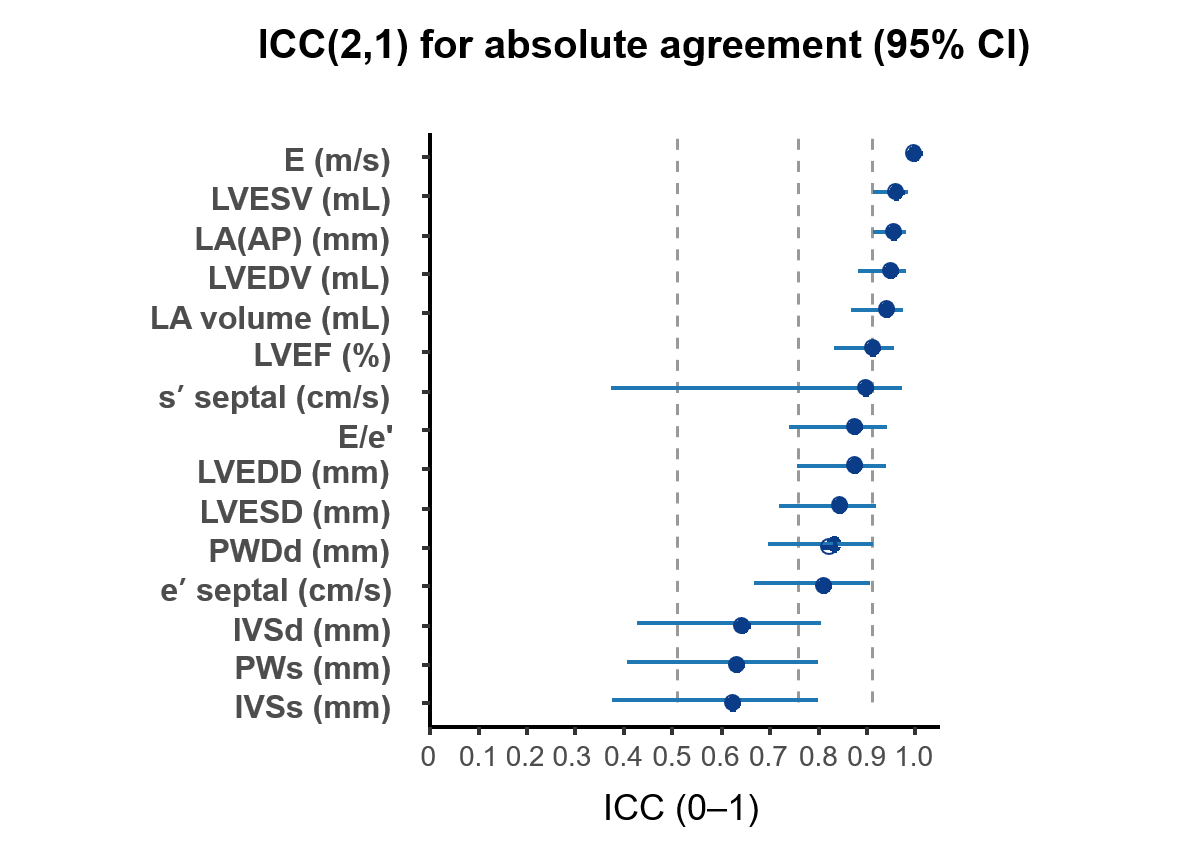
**

| **Variable** | **N** | **ICC(2,1) (95% CI)** | **Med AE [Q1–Q3]** | **Mean Δ (AI–RS)** |
| --- | --- | --- | --- | --- |
| **E(m/s)** | 39 | 0.989 (0.972–0.995) | 0.03 [0.02–0.05] | -0.02 |
| **LVESV (ml)** | 40 | 0.950 (0.903–0.974) | 3.20 [1.28–5.33] | -2.77 |
| **LA(AP) (mm)** | 40 | 0.947 (0.902–0.971) | 1.05 [0.40–2.28] | -0.37 |
| **LVEDV (ml)** | 40 | 0.940 (0.871–0.970) | 6.30 [3.02–15.75] | -6.39 |
| **LA vol (ml)** | 40 | 0.933 (0.857–0.966) | 14.00 [8.52–22.18] | -9.02 |
| **LVEF** | 40 | 0.902 (0.822–0.947) | 2.53 [1.02–4.57] | -0.00 |
| **s' septal (cm/s)** | 39 | 0.888 (0.361–0.964) | 0.50 [0.25–0.80] | -0.56 |
| **E/e'** | 39 | 0.866 (0.729–0.932) | 2.09 [0.99–4.00] | 1.55 |
| **LVEDD (mm)** | 40 | 0.865 (0.747–0.928) | 1.87 [1.04–3.72] | -1.40 |
| **LVESD (mm)** | 40 | 0.834 (0.708–0.908) | 2.35 [0.93–5.25] | -1.24 |
| **PWDd (mm)** | 40 | 0.822 (0.688–0.902) | 0.96 [0.38–1.78] | -0.06 |
| **e' septal (cm/s)** | 39 | 0.811 (0.657–0.898) | 0.90 [0.60–1.30] | -0.46 |
| **IVSd (mm)** | 39 | 0.644 (0.415–0.796) | 1.32 [0.58–2.16] | 0.65 |
| **PWs (mm)** | 40 | 0.633 (0.397–0.789) | 1.01 [0.51–1.88] | 0.88 |
| **IVSs (mm)** | 40 | 0.625 (0.366–0.789) | 1.14 [0.59–2.35] | 0.89 |

Values are presented for variables with ≥30 paired measurements available. AE, absolute error (|AI − reference standard|); AI, artificial intelligence; CI, confidence interval; E, early mitral inflow velocity; E/e′, ratio of early mitral inflow velocity to early diastolic septal mitral annular velocity; e′ septal, early diastolic septal mitral annular velocity; ICC, intraclass correlation coefficient for absolute agreement; IVSd, interventricular septal thickness at end-diastole; IVSs, interventricular septal thickness at end-systole; LA(AP), left atrial anteroposterior dimension; LA vol, left atrial volume; LVEDV, left ventricular end-diastolic volume; LVEDD, left ventricular end-diastolic dimension; LVEF, left ventricular ejection fraction; LVESD, left ventricular end-systolic dimension; LVESV, left ventricular end-systolic volume; Med, median; PWDd, posterior wall thickness at end-diastole; PWs, posterior wall thickness at end-systole; Q1–Q3, first to third quartile; RS, reference standard; s′ septal, peak systolic septal mitral annular velocity; Δ, mean difference (AI − reference standard).

**Supplementary Results 2. Class-wise diagnostic performance for aortic regurgitation grading**

- 1. **AI versus Reference Standard**

|  | **Accuracy** | **Sensitivity** | **Specificity** | **PPV** | **NPV** |
| --- | --- | --- | --- | --- | --- |
| **None/Trivial** | 0.950 | 1.000 | 0.905 | 0.905 | 1.000 |
| **Mild** | 0.825 | 0.900 | 0.800 | 0.600 | 0.960 |
| **Moderate** | 0.775 | 0.000 | 0.969 | 0.000 | 0.795 |
| **Severe** | 0.950 | 0.667 | 0.973 | 0.667 | 0.973 |

Values are calculated on a one-vs-rest basis for each AR severity category (N=40) using the cardiologist-adjudicated reference standard. AR, aortic regurgitation; NPV, negative predictive value; PPV, positive predictive value.

- 1. **Human versus Reference Standard**

|  | **Accuracy** | **Sensitivity** | **Specificity** | **PPV** | **NPV** |
| --- | --- | --- | --- | --- | --- |
| **None/Trivial** | 0.950 | 0.947 | 0.952 | 0.947 | 0.952 |
| **Mild** | 0.825 | 0.600 | 0.900 | 0.667 | 0.871 |
| **Moderate** | 0.875 | 0.750 | 0.906 | 0.667 | 0.935 |
| **Severe** | 1.000 | 1.000 | 1.000 | 1.000 | 1.000 |

Values are calculated on a one-vs-rest basis for each AR severity category (N=40) using the cardiologist-adjudicated reference standard. AR, aortic regurgitation; NPV, negative predictive value; PPV, positive predictive value.

**Supplementary Results 3. Confusion matrices for additional qualitative grading (mitral regurgitation, tricuspid regurgitation, and pericardial effusion)**

**3.1 Mitral regurgitation (MR)**

**
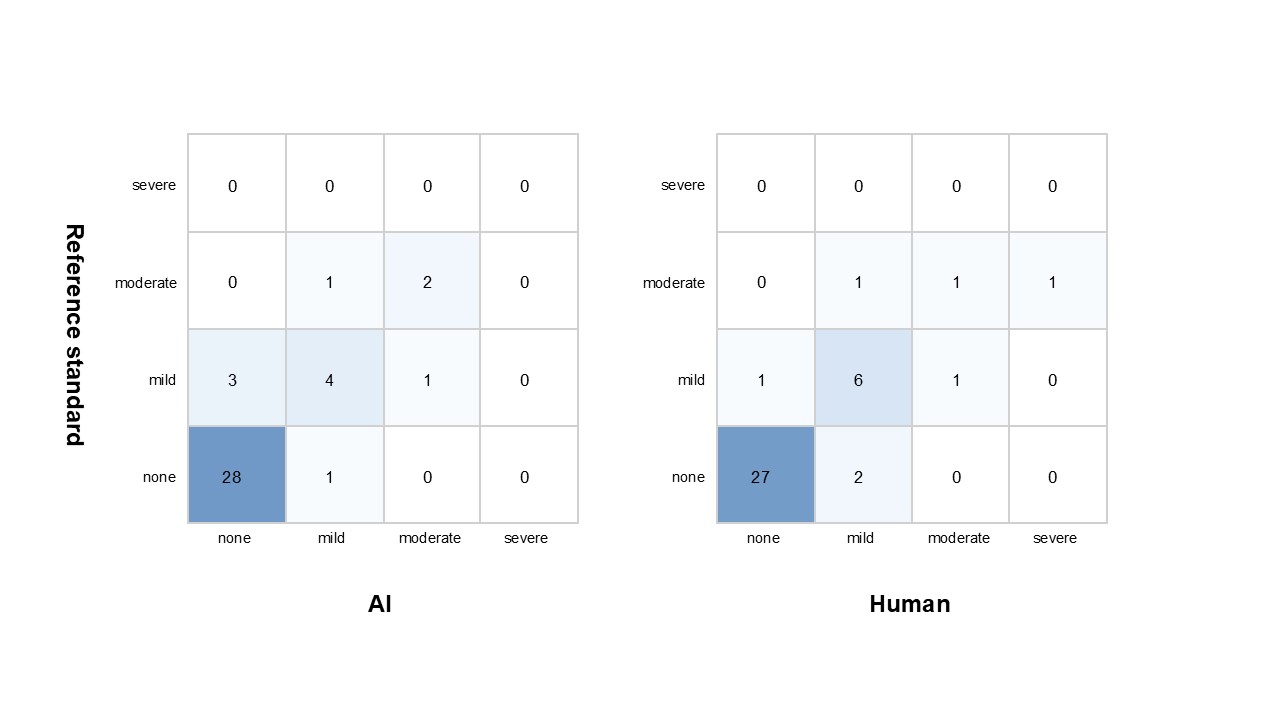
**

**3.2. Tricuspid regurgitation (TR)**

**
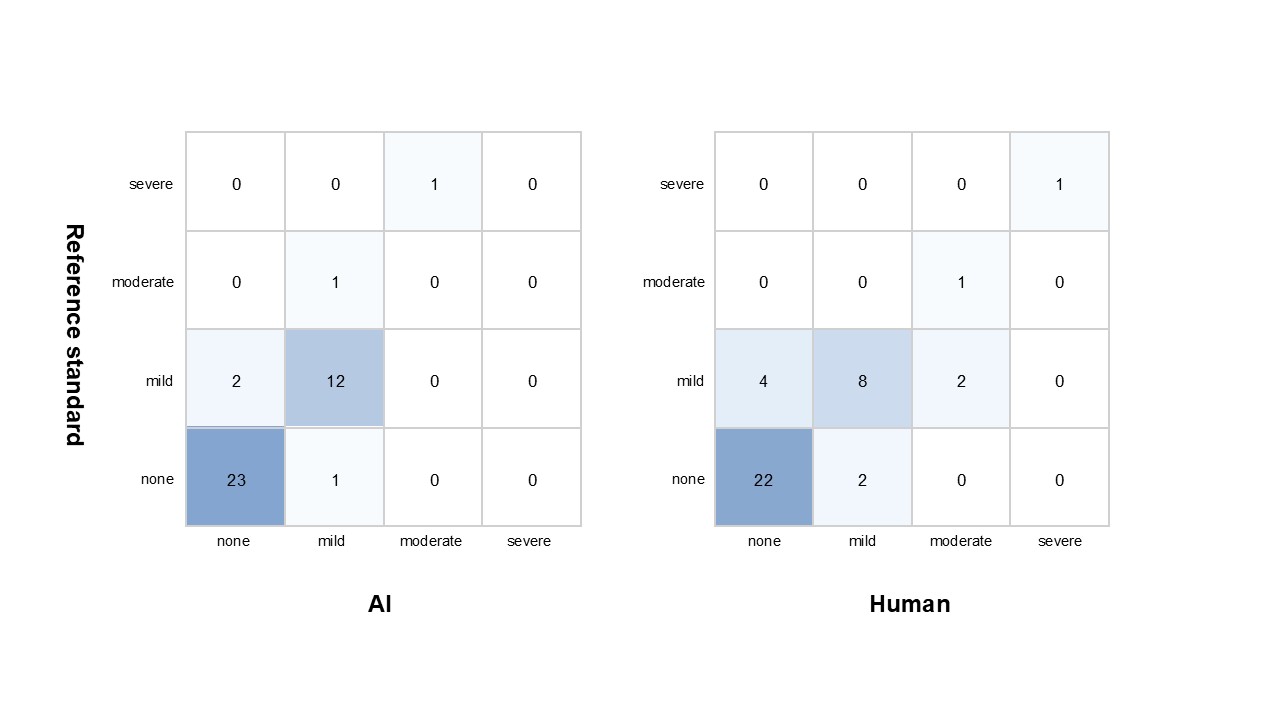
**

**3.3 Pericardial effusion**

**
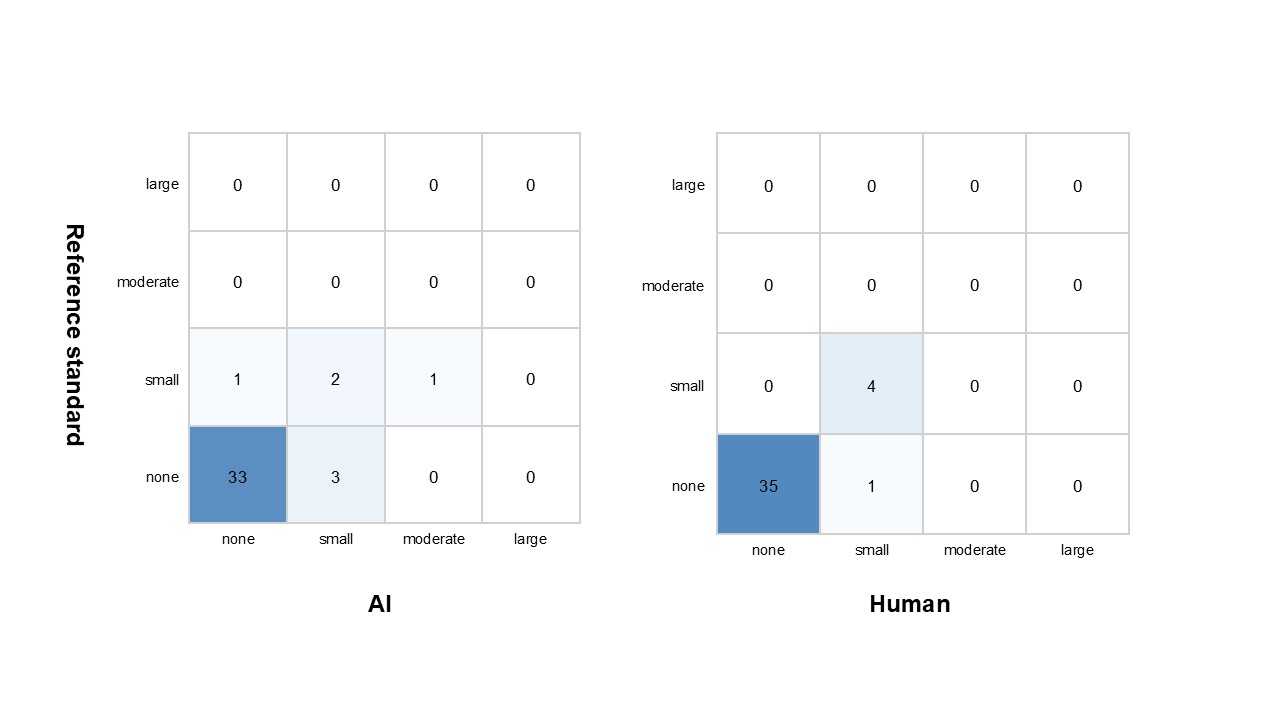
**
